# Chitosan–Curcumin Bioactive Platforms: Mechanistic Synergy, Antimicrobial Performance, and Design Principles for Next-Generation Wound Therapies

**DOI:** 10.3390/polym18111329

**Published:** 2026-05-28

**Authors:** Moorthy Maruthapandi, John H. T. Luong

**Affiliations:** 1 Department of Chemistry, Bar-Ilan University, Ramat-Gan 52900, Israel; 2 Bar-Ilan Institute for Nanotechnology and Advanced Material, Bar-Ilan University, Ramat-Gan 52900, Israel; 3 School of Chemistry, University College Cork, T12 YN60 Cork, Ireland

**Keywords:** chitosan–curcumin systems, multifunctional biomaterials, antimicrobial and antifungal activity, biofilm disruption, wound healing, nanoparticle delivery platforms, solubility and bioavailability enhancement, Mechanistic synergy, polymicrobial infections, artificial intelligence-guided formulation

## Abstract

Chronic and infected wounds remain difficult to treat due to persistent microbial burden, biofilm formation, and dysregulated inflammation. As a multifunctional polyphenol, curcumin exhibits broad-spectrum antimicrobial, anti-inflammatory, and antioxidant activities. Nevertheless, the clinical application of curcumin is constrained by its limited solubility in water, inherent instability, and insufficient bioavailability. Chitosan, a cationic polysaccharide, provides complementary advantages including intrinsic antimicrobial activity, mucoadhesion, and the capacity to form versatile delivery platforms such as nanoparticles, hydrogels, and films. This review reframes chitosan–curcumin systems as dual-function bioactive platforms in which both the carrier and payload actively contribute to therapeutic outcomes. Mechanistically, chitosan disrupts microbial membranes, enhances bioadhesion, and supports tissue regeneration, while curcumin modulates intracellular targets including reactive oxygen species, quorum sensing, and inflammatory signaling pathways. Their integration enables multimodal antimicrobial activity, improved biofilm disruption, and coordinated regulation of the wound-healing cascade. This review critically examines the structure–function relationships governing release kinetics, stability, and cytocompatibility, with particular emphasis on chitosan molecular weight, degree of deacetylation, crosslinking strategies, and curcumin loading. Solubility-enhancement strategies for curcumin, including surfactants, nanoparticles, solid dispersions, and chemical derivatives, are evaluated in the context of antimicrobial efficacy and cytotoxicity. Finally, the review highlights translational challenges and future directions, such as antibiotic synergy, antifungal applications, formulation complexity, and the emerging role of artificial intelligence in predictive material design. Collectively, these insights establish design principles for next-generation multifunctional biomaterials that integrate antimicrobial activity with immune modulation and tissue repair.

## 1. Introduction

Chronic and infected wounds represent a persistent and escalating clinical challenge, driven by the convergence of microbial colonization, biofilm formation, impaired vascularization, and dysregulated inflammatory responses [1]. Despite advancements in wound care, these lesions frequently fail to progress through physiological healing stages, resulting in prolonged morbidity and a substantial healthcare burden [2,3]. Antibiotic-resistant pathogens make treatment difficult because standard drugs often work poorly due to limited tissue penetration, biofilm-related resistance, and the complexity of wound environments [4,5,6].

**Figure 1:**
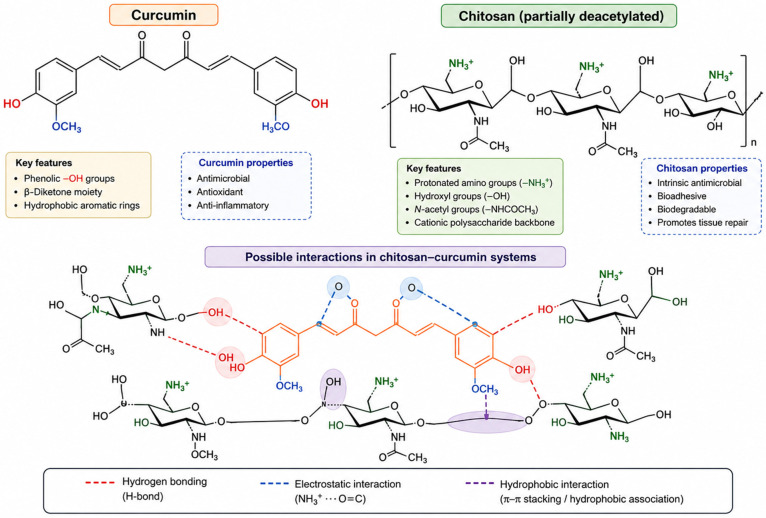
(**Upper left**) Curcumin (C_21_H_20_O_6_, MW = 368.4 g/mol, aqueous solubility of curcumin is typically below 0.01 mg/mL at physiological pH). (**Upper right**) Chemical structure of chitosan, a linear polysaccharide composed of randomly distributed β-(1→4)-linked D-glucosamine (deacetylated unit) and N-acetyl-D-glucosamine (acetylated unit). The integration of curcumin into chitosan matrices is postulated to occur through a combination of several interactions (**Lower**).

Natural bioactive compounds have emerged as promising alternatives or adjuncts to traditional antimicrobials. Among these, curcumin (Figure 1, upper, left), a polyphenolic constituent derived from *Curcuma longa*, has garnered significant attention for its broad-spectrum antimicrobial, anti-inflammatory, and antioxidant activities [7,8,9]. Curcumin disrupts microbial membranes, interferes with intracellular signaling pathways, and modulates key inflammatory mediators, including the nuclear factor kappa-light-chain-enhancer of activated B cells (NF-κB) and various pro-inflammatory cytokines [9,10]. These attributes position curcumin as a multifunctional therapeutic agent capable of simultaneously addressing infection and inflammation. However, its clinical translation remains constrained by poor aqueous solubility, rapid degradation, and limited bioavailability [11,12,13].

In parallel, chitosan (Figure 1, upper, right), a cationic polysaccharide derived from chitin [14,15], has been extensively investigated as a biomaterial for antimicrobial and wound-healing applications [16,17,18]. Its intrinsic antimicrobial activity originates from electrostatic interactions with negatively charged microbial membranes, leading to structural disruption and the leakage of intracellular constituents [19,20]. Beyond its antimicrobial properties, chitosan exhibits excellent biocompatibility, mucoadhesion, and the capacity to form versatile delivery systems, including nanoparticles, hydrogels, and films [17,18,21]. Notably, chitosan-based scaffolds can support hemostasis, promote cell adhesion, and facilitate tissue regeneration, rendering them particularly advantageous for wound management [22,23].

Curcumin, with phenolic hydroxyl groups, a β-diketone moiety, and conjugated aromatic rings (Figure 1, upper-left), enables multiple non-covalent interactions with chitosan (Figure 1, lower). The integration of curcumin into chitosan matrices is postulated to occur through the following routes:

- Hydrogen bonding between hydroxyl, amino, and carbonyl groups;
- Hydrophobic associations involving the aromatic domains of curcumin;
- π–π stacking interactions between conjugated structures;
- Possible electrostatic interactions between protonated amino groups of chitosan and electron-rich oxygen-containing functionalities of curcumin.

**Figure 2:**
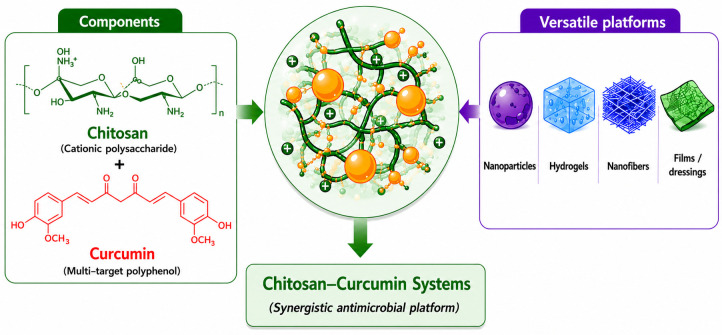
Schematic representation of chitosan–curcumin systems as multifunctional antimicrobial and wound-healing platforms. (**Left**): chemical structures and principal characteristics of chitosan and curcumin, highlighting the cationic polysaccharide nature of chitosan and the polyphenolic structure of curcumin. (**Center**): conceptual illustration of chitosan (green)–curcumin (orange) interactions and self-assembled composite formation through electrostatic interactions, hydrogen bonding, and hydrophobic associations, leading to integrated multifunctional biomaterial systems. (**Right**): representative delivery and formulation platforms, including nanoparticles, hydrogels, nanofibers, and films/dressings, developed for antimicrobial, antibiofilm, and tissue-regenerative applications.

These interactions facilitate the formation of integrated chitosan–curcumin systems with improved curcumin stability, dispersion, encapsulation efficiency, and controlled-release behavior. The resulting multifunctional platforms can be engineered into diverse formulations, including nanoparticles, hydrogels, nanofibers, and films/dressings for antimicrobial, antibiofilm, and wound-healing applications (Figure 2). These interactions enhance curcumin stability, dispersion, encapsulation, and release, improving the antimicrobial, antibiofilm, antioxidant, and wound-healing performance of chitosan–curcumin biomaterials.

The integration of curcumin within chitosan matrices offers a compelling strategy to mitigate the limitations of each component while leveraging their complementary mechanisms. In such systems, chitosan functions not only as a delivery vehicle that enhances the stability and bioavailability of curcumin but also as an active participant in antimicrobial and regenerative processes [24]. Concurrently, curcumin provides intracellular antimicrobial activity and immunomodulatory effects that extend beyond membrane-level interactions [9,10]. This combination facilitates a multimodal therapeutic approach, targeting microbial viability, biofilm integrity, inflammatory signaling, and tissue repair in a coordinated manner.

In this context, chitosan–curcumin formulations should be conceptualized as dual-function bioactive platforms rather than mere drug-delivery systems, as both the carrier and the payload independently and synergistically contribute to therapeutic outcomes. This paradigm shift is critical for advancing the design of next-generation antimicrobial biomaterials tailored to the complexity of infected and chronic wounds [25].

This review provides a comprehensive, mechanistically grounded analysis of chitosan–curcumin systems. It first examines the individual biological functions and limitations of curcumin and chitosan, followed by an evaluation of their synergistic interactions. Subsequently, the review discusses current delivery architectures and highlights key structure–function relationships governing antimicrobial activity, released kinetics, and cytocompatibility. Finally, translational challenges and delineated design principles will be addressed for the development of clinically relevant, multifunctional biomaterials.

For this manuscript, the literature was identified through structured searches of PubMed, Web of Science, Scopus, and Google Scholar, supplemented by manual screening of reference lists. The primary search window covered 2020–2026, with earlier foundational studies included when relevant. Keywords included “curcumin chitosan,” “curcumin-loaded chitosan nanoparticles,” “curcumin wound healing,” “chitosan antimicrobial,” and related terms. Studies were included if they examined curcumin–chitosan formulations with antimicrobial, antibiofilm, antioxidant, or wound-healing relevance; studies lacking primary data or focusing on unrelated applications including anti-cancer were excluded. This approach ensures that the review is comprehensive, transparent, and free from selection bias.

Despite the individual research fields of chitosan-based biomaterials and curcumin-loaded delivery systems both being extensive, the number of studies that explicitly investigate their combined use remains relatively limited. This reflects the emerging nature of chitosan–curcumin platforms rather than a lack of scientific merit. Indeed, the available studies—spanning nanoparticles, nanofibers, hydrogels, and composite dressings—consistently demonstrate enhanced antimicrobial activity, improved curcumin stability, superior biofilm disruption, and accelerated wound healing compared with either component alone. The growing body of work, including several recent reports, provides a coherent mechanistic rationale for the observed synergy and highlights the translational potential of these hybrid systems. As such, even though the literature is still developing, the convergence of findings across diverse formulations supports the robustness of the conclusions presented in this review.

## 2. Curcumin: Antimicrobial Mechanisms and Biological Functions

Bacterial infections are a common problem associated with wound treatment that imposes a significant burden on healthcare systems and patients. As a result, healthcare providers urgently need new treatment strategies to protect people. Hydrogel biomaterials with inherent antimicrobial properties offer an attractive and viable solution to this issue. Here, for the first time, we have developed a new efficient synthetic strategy to prepare cationic hydrogels (PHCI) with intrinsically efficient antimicrobial properties by chemically cross-linking *trans*-1,4-cyclohexanediamine with 1,3-dibromo-2-propanol using a condensation reaction without the use of toxic cross-linking agents. As expected, the prepared PHCI hydrogel possessed an inherent antibacterial ability that can adsorb and kill *Staphylococcus aureus* and *Escherichia coli* electrostatically. Notably, in vivo experiments on normal and diabetic rat models confirmed that the PHCI hydrogel can quickly stop bleeding, efficiently kill bacteria, promote the conversion of macrophages from the proinflammatory M1 phenotype to the repaired M2 phenotype, and accelerate collagen deposition and blood vessel formation, thereby achieving rapid wound healing. Overall, this work presents an effective antibacterial dressing that might provide a facile but effective approach for clinical wound management.

Curcumin, a hydrophobic polyphenol derived from *Curcuma longa*, has been extensively investigated for its broad-spectrum antimicrobial, anti-inflammatory, and antioxidant activities [26]. Its pleiotropic biological effects arise from its ability to interact with multiple cellular targets, enabling the simultaneous modulation of microbial viability, oxidative stress, and host inflammatory responses [7,8]. Unlike conventional antibiotics that typically act on specific molecular targets, curcumin exerts multimodal antimicrobial activity, which contributes to its reduced propensity for inducing resistance [9,27].

### 2.1. Antimicrobial Mechanisms

#### 2.1.1. Membrane Disruption and Permeability Alteration

One of the primary antimicrobial actions of curcumin involves direct interaction with microbial cell membranes [28]. Curcumin can insert into lipid bilayers, altering membrane integrity and increasing permeability, which leads to the leakage of intracellular components and eventual cell death [9,27]. This effect has been observed in both Gram-positive and Gram-negative bacteria, although differences in cell wall structure influence susceptibility. In Gram-negative bacteria, the outer membrane may partially limit curcumin penetration; however, once internalized, curcumin disrupts membrane potential and compromises cellular homeostasis.

#### 2.1.2. Intracellular Targeting and Metabolic Interference

Beyond membrane-level interactions, curcumin exerts antimicrobial effects through interference with intracellular processes. It inhibits key enzymes involved in DNA replication, transcription, and protein synthesis, thereby impairing bacterial growth and survival [7,29]. Curcumin can also bind to bacterial proteins and nucleic acids, disrupting essential metabolic pathways [30]. These intracellular effects are particularly important in preventing bacterial recovery following initial membrane damage.

#### 2.1.3. Reactive Oxygen Species (ROS) Modulation

Curcumin exhibits complex redox behavior, functioning as both an antioxidant and a pro-oxidant depending on the microenvironment [31]. In microbial systems, curcumin can induce the generation of ROS, leading to oxidative damage of cellular components, including lipids, proteins, and DNA [9,32]. This oxidative stress contributes to microbial cell death and enhances the overall antimicrobial effect. In contrast, within host tissues, curcumin often acts as an antioxidant, scavenging excess ROS and protecting cells from oxidative damage, thereby supporting tissue repair.

#### 2.1.4. Biofilm Inhibition and Quorum Sensing Interference

Biofilm formation represents a major barrier to effective antimicrobial therapy. Curcumin has been shown to inhibit biofilm formation and disrupt established biofilms by interfering with quorum sensing pathways and extracellular polymeric substance (EPS) production [33,34]. By modulating signaling molecules involved in bacterial communication, curcumin reduces biofilm stability and enhances susceptibility to antimicrobial agents. This property is particularly relevant for chronic wound infections, where biofilms contribute to persistent inflammation and delayed healing.

### 2.2. Anti-Inflammatory and Immunomodulatory Effects

In addition to its direct antimicrobial activity, curcumin plays a critical role in modulating host inflammatory responses. It suppresses key signaling pathways, including NF-κB, thereby reducing the expression of pro-inflammatory cytokines such as tumor necrosis factor (TNF)-α, interleukin (IL)-1β, and IL-6 [7,10]. Curcumin also influences macrophage polarization, promoting a shift from pro-inflammatory (M1) to anti-inflammatory (M2) phenotypes [35], which is essential for the resolution of inflammation and progression of wound healing. Furthermore, curcumin can modulate the activity of various immune cells, including neutrophils, dendritic cells, and T lymphocytes, contributing to a balanced immune response [8].

### 2.3. Antioxidant Activity and Tissue Protection

Curcumin is a well-established antioxidant capable of scavenging reactive oxygen and nitrogen species, as well as upregulating endogenous antioxidant enzymes such as superoxide dismutase (SOD), catalase, and glutathione peroxidase [32]. By reducing oxidative stress, curcumin protects cellular components from damage and supports tissue repair processes. This antioxidant activity is especially important in wound environments characterized by elevated ROS levels and oxidative imbalance.

### 2.4. Limitations of Curcumin in Biomedical Applications

Despite its promising biological activities, curcumin faces significant challenges that limit its clinical translation. Its poor aqueous solubility results in low absorption and limited bioavailability [12,13]. Additionally, curcumin is chemically unstable under physiological conditions, undergoing rapid degradation and metabolic transformation [36]. These limitations result in insufficient therapeutic concentrations at target sites and reduced in vivo efficacy. Moreover, rapid clearance and a lack of targeted delivery further hinder its performance in complex biological environments. These challenges necessitate the development of effective delivery systems that can enhance curcumin stability, control its release, and improve localization at sites of infection.

### 2.5. Solubility and Bioavailability Enhancement of Curcumin

Curcumin’s very limited aqueous solubility (<0.01 mg/mL at physiological pH) remains a primary barrier to its therapeutic use. Numerous strategies have been developed to enhance solubility, stability, and bioavailability, each with distinct implications for cytotoxicity and antimicrobial performance [37].

Chemical modification is a direct strategy; water-soluble curcumin derivatives—such as PEGylated conjugates or alkyl-sulfonate analogues exhibit markedly improved solubility and enhanced antioxidant activity [38,39]. However, increased solubility often correlates with higher cytotoxicity, necessitating dose optimization. Surfactant-based systems and biosurfactants, such as surfactin, improve dissolution through micellar solubilization, though they may introduce membrane-disruptive cytotoxicity at higher concentrations [40,41].

Nanoparticle-based strategies, including polymeric nanoparticles and protein complexes (e.g., cod-protein nanoparticles), offer controlled release and improved stability [42]. Nanoformulations generally increase antimicrobial efficacy by improving membrane penetration and intracellular accumulation [43,44]. Emerging approaches, such as deep eutectic solvent (DES) microemulsions and hydrophilic polymer solid dispersions, dramatically increase dissolution rates and can potentiate conventional antimicrobials [45,46]. For chitosan–curcumin systems, selecting an appropriate solubilization method requires balancing enhanced bioavailability with safety, stability, and physicochemical compatibility with the chitosan matrix.

## 3. Chitosan as a Functional Antimicrobial and Delivery Platform

Chitosan, a cationic polysaccharide derived from the partial deacetylation of chitin, has emerged as a versatile biomaterial for antimicrobial and biomedical applications. Its unique combination of physicochemical properties, including biodegradability, biocompatibility, and intrinsic antimicrobial activity, has led to widespread investigation in drug delivery, tissue engineering, and wound management [16,17]. Unlike inert polymeric carriers, chitosan actively participates in biological processes, rendering it particularly attractive for the development of multifunctional therapeutic systems.

### 3.1. Intrinsic Antimicrobial Activity

#### 3.1.1. Electrostatic Interactions and Membrane Disruption

The antimicrobial activity of chitosan is primarily attributed to its polycationic nature, arising from protonated amino groups under acidic and physiological conditions. These positively charged moieties interact with negatively charged components of microbial cell membranes, leading to the disruption of membrane integrity, increased permeability, and the subsequent leakage of intracellular constituents [17,20]. This mechanism is effective against a broad spectrum of microorganisms, including Gram-positive and Gram-negative bacteria, as well as various fungi. The magnitude of this antimicrobial effect is strongly influenced by physicochemical parameters such as molecular weight, degree of deacetylation (DDA), and environmental pH. While higher charge density generally enhances membrane interaction, excessive charge may also increase cytotoxicity toward mammalian cells.

#### 3.1.2. Interaction with Intracellular Targets

Beyond membrane-level effects, chitosan can penetrate microbial cells and interact with intracellular targets, including DNA and proteins. This interaction may inhibit transcription and protein synthesis, further impairing microbial viability [19]. Low-molecular-weight chitosan and chitosan oligomers are particularly effective in this regard due to their enhanced capacity to traverse cellular membranes.

#### 3.1.3. Metal Chelation and Nutrient Deprivation

Chitosan possesses the ability to chelate metal ions and essential nutrients, thereby depriving microorganisms of critical elements required for growth and metabolism [47]. This indirect antimicrobial mechanism contributes to its overall efficacy and may act synergistically with direct membrane disruption and intracellular interference.

### 3.2. Bioadhesion and Mucoadhesive Properties

A defining characteristic of chitosan is its robust bioadhesive behavior, which originates from electrostatic interactions with negatively charged biological surfaces, including mucosal tissues and extracellular matrix components [48]. This property enhances the retention of chitosan-based formulations at target sites, improving local drug concentrations and therapeutic efficacy. In wound environments, this adhesion facilitates intimate contact with the wound bed, promoting localized antimicrobial action and supporting tissue regeneration. Furthermore, it enables chitosan to function as a protective barrier, shielding the wound from external contamination while maintaining a moist healing environment.

### 3.3. Film-Forming and Scaffold Properties

Chitosan exhibits excellent film-forming capabilities, allowing for the fabrication of membranes, coatings, and wound dressings with tunable mechanical and permeability properties [23]. These materials can serve as physical barriers while simultaneously delivering therapeutic agents. In addition to films, chitosan can be processed into hydrogels, sponges, and porous scaffolds that mimic the architecture of the natural extracellular matrix. These structures support cell adhesion, proliferation, and migration, thereby facilitating tissue repair [18]. The ability to tailor porosity, mechanical strength, and degradation rates makes chitosan highly adaptable to diverse wound types and clinical requirements.

### 3.4. Role in Wound Healing and Tissue Regeneration

In addition to its antimicrobial properties, chitosan plays an active role in modulating the wound-healing cascade. It has been shown to promote hemostasis by accelerating platelet aggregation and clot formation, which is critical during the early stages of repair [22]. Chitosan also supports fibroblast proliferation, collagen deposition, and angiogenesis, contributing to the formation of new tissue. Importantly, chitosan can modulate inflammatory responses, helping to balance the transition from the inflammatory phase to the proliferative phase of healing. This immunomodulatory effect is particularly beneficial in chronic wounds, where prolonged inflammation often impedes regeneration.

### 3.5. Versatility as a Drug Delivery Platform

Chitosan’s chemical structure permits facile modification and functionalization, enabling the development of diverse delivery systems, including nanoparticles, nanofibers, hydrogels, and composite materials [17,49]. These systems can encapsulate and protect bioactive compounds, control release kinetics, and enhance stability under physiological conditions. In nanoparticle form, chitosan can improve the solubility and bioavailability of hydrophobic compounds, while its surface charge facilitates interaction with biological membranes and enhances cellular uptake. In hydrogel and film formats, chitosan enables the sustained and localized release of therapeutic agents, making it exceptionally suitable for wound dressing applications.

The multifunctional nature of chitosan as both an antimicrobial agent and a versatile delivery platform provides a rigorous foundation for the integration of bioactive molecules such as curcumin. By combining chitosan’s membrane-targeting and scaffold-forming capabilities with curcumin’s intracellular and immunomodulatory effects, it is possible to design dual-function systems with enhanced therapeutic performance.

## 4. Mechanistic Synergy in Chitosan–Curcumin Systems

**Figure 3:**
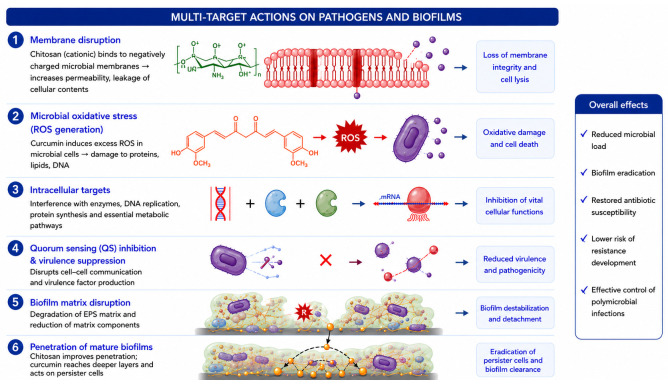
Proposed multi-target antimicrobial and antibiofilm mechanisms of chitosan–curcumin systems against microbial pathogens and mature biofilms. In the schematic, green structures represent chitosan-related components (**first row**), orange structures (**second row**) indicate curcumin-related components, purple structures denote microbial cells and released cellular contents, and red symbols indicate ROS-associated oxidative stress (**second row**). In the (**third row**), the “intracellular targets” panel schematically illustrates interference with essential microbial intracellular processes, including enzyme activity, DNA replication, transcription, and protein synthesis. The blue and green shapes represent intracellular enzymatic or metabolic targets, whereas the following red structure represents disruption of mRNA-associated protein synthesis machinery. Dashed and directional arrows represent proposed mechanistic interactions and downstream biological effects (**last row**).

The integration of chitosan and curcumin facilitates the development of dual-function bioactive systems in which both components contribute independently and synergistically to antimicrobial activity, immunomodulation, and tissue regeneration. Unlike conventional drug delivery approaches that rely on inert carriers, chitosan–curcumin systems exhibit multimodal and orthogonal mechanisms of action, targeting distinct yet complementary biological pathways (Figure 3). This synergy enhances therapeutic efficacy while mitigating the likelihood of resistance development and treatment failure.

### 4.1. Membrane–Intracellular Synergy

A defining feature of chitosan–curcumin systems is the integration of membrane-targeting and intracellular mechanisms. Chitosan, owing to its polycationic nature, interacts with negatively charged microbial cell membranes, leading to membrane destabilization, increased permeability, and the leakage of intracellular contents [19,20]. This initial disruption compromises membrane integrity and facilitates the penetration of co-delivered agents.

Curcumin, in contrast, exerts its primary antimicrobial effects within the cell by interfering with enzyme activity, nucleic acid function, and metabolic pathways, while simultaneously inducing oxidative stress through reactive oxygen species (ROS) generation [9,32]. When combined with chitosan, the enhanced permeability of microbial membranes promotes increased intracellular accumulation of curcumin, thereby amplifying its antimicrobial potency. This sequential and complementary mechanism—membrane disruption followed by intracellular interference—represents a critical advantage over single-agent systems. By simultaneously targeting multiple cellular compartments, chitosan–curcumin systems reduce the capacity of microorganisms to adapt or develop resistance.

### 4.2. Biofilm Disruption and Penetration Synergy

Biofilms present a significant barrier to antimicrobial treatment due to their dense extracellular polymeric substance (EPS) matrix and the reduced metabolic activity of embedded cells [50]. Chitosan–curcumin systems exhibit enhanced antibiofilm activity through combined physicochemical and biochemical mechanisms. Chitosan contributes to biofilm disruption by interacting with negatively charged EPS components, leading to structural destabilization and increased matrix permeability [20,47]. Its bioadhesive properties facilitate intimate contact with the biofilm surface, enabling sustained local activity.

Curcumin further enhances antibiofilm efficacy by interfering with quorum sensing pathways and reducing the expression of genes associated with biofilm formation and maintenance [33,34]. Additionally, curcumin-mediated ROS generation contributes to the degradation of biofilm components and microbial cells. The combination of matrix disruption (chitosan) and signaling interference (curcumin) enables deep penetration and the effective eradication of biofilms, which is particularly relevant in chronic wound infections, where biofilms drive persistence and delayed healing.

### 4.3. Anti-Inflammatory and Immunomodulatory Synergy

Chronic wounds are characterized by prolonged and dysregulated inflammation, which impairs tissue repair. Chitosan–curcumin systems address this challenge through complementary immunomodulatory mechanisms. Curcumin is a potent regulator of inflammatory signaling pathways, particularly through the inhibition of NF-κB, resulting in the reduced expression of pro-inflammatory cytokines such as TNF-α, IL-1β, and IL-6 [7,10]. It also promotes macrophage polarization toward the anti-inflammatory M2 phenotype, facilitating the resolution of inflammation.

Chitosan contributes to immune modulation by influencing cellular responses at the wound site, including the activation of macrophages, stimulation of growth factor release, and enhancement of tissue remodeling processes [18,22]. Together, these effects result in the coordinated regulation of inflammation, reducing excessive immune responses while promoting regenerative processes.

### 4.4. Integration of Antimicrobial Activity with Wound Healing

A major limitation of many antimicrobial agents is their inability to support tissue regeneration. In contrast, chitosan–curcumin systems integrate antimicrobial activity with wound-healing functions, addressing both infection control and tissue repair. Chitosan provides a structural framework that supports cell adhesion, proliferation, and migration, while also promoting hemostasis and angiogenesis [22,23]. Its scaffold-forming capability enables the creation of matrices that mimic the extracellular matrix. Curcumin complements these effects by reducing oxidative stress and modulating inflammatory signaling, thereby creating a microenvironment conducive to healing [8,32].

**Figure 4:**
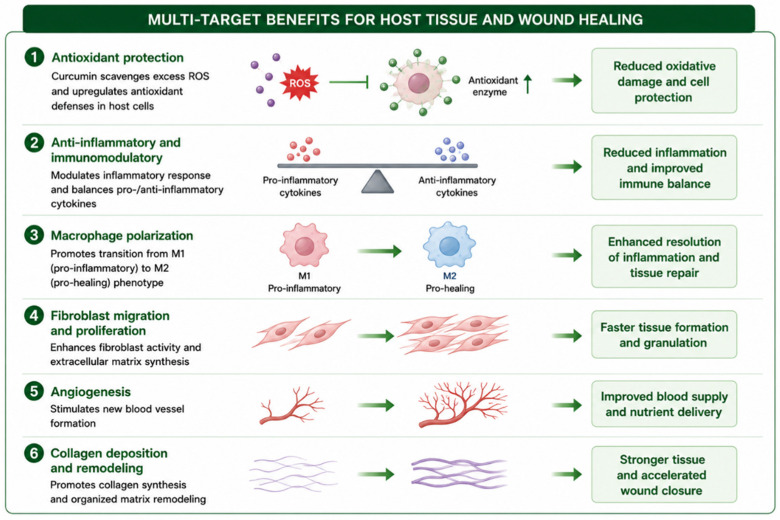
Proposed host-protective and wound-healing mechanisms of chitosan–curcumin systems. Beyond their combined antimicrobial activities, chitosan–curcumin platforms contribute to tissue repair through multiple complementary biological pathways. Curcumin scavenges excess ROS and enhances endogenous antioxidant defenses, thereby reducing oxidative stress-mediated cellular damage. The combined system also modulates inflammatory responses by balancing pro-inflammatory and anti-inflammatory cytokine signaling and promoting macrophage polarization from the pro-inflammatory M1 phenotype toward the pro-healing M2 phenotype. Furthermore, chitosan–curcumin systems support fibroblast migration and proliferation, stimulate angiogenesis and neovascularization, and promote collagen deposition and extracellular matrix remodeling. Collectively, these integrated effects facilitate improved immune balance, accelerated granulation tissue formation, enhanced tissue regeneration, and faster wound closure. Green arrows indicate proposed beneficial biological progression or enhancement of wound-healing responses, whereas color-coded cellular and molecular illustrations represent distinct host-related processes, including oxidative stress modulation, cytokine balancing, macrophage polarization, fibroblast activity, angiogenesis, and collagen remodeling.

Beyond direct antimicrobial and antibiofilm activity, chitosan–curcumin systems exert important protective and regenerative effects on host tissues, which are particularly relevant for chronic and infected wounds. Persistent oxidative stress, dysregulated inflammation, impaired angiogenesis, and defective extracellular matrix remodeling are hallmark features of non-healing wounds. Curcumin contributes through potent antioxidant and anti-inflammatory activities, including ROS scavenging, modulation of cytokine signaling, and regulation of macrophage polarization, while chitosan provides a supportive bioactive matrix that promotes fibroblast proliferation, collagen deposition, and tissue regeneration. The integration of these complementary mechanisms enables chitosan–curcumin systems to function not only as antimicrobial platforms but also as multifunctional wound-healing biomaterials capable of coordinating tissue repair and immune modulation (Figure 4).

### 4.5. Kinetic and Delivery Synergy

Chitosan-based matrices encapsulate curcumin, protecting it from degradation and improving its stability under physiological conditions [49,50,51]. This encapsulation enables controlled and sustained release, maintaining therapeutic concentrations over extended periods. The physicochemical properties of chitosan—such as molecular weight, degree of deacetylation, and crosslinking density determine release kinetics and interaction with biological tissues. Furthermore, the bioadhesive nature of chitosan enhances retention at target sites, reducing systemic loss and improving localized efficacy.

## 5. Design Principles and Structure–Function Relationships

The performance of chitosan–curcumin systems is governed by a complex interplay of physicochemical and biological parameters. Unlike conventional formulations, where the carrier serves a passive role, these platforms require the co-optimization of both carrier and payload.

### 5.1. Influence of Chitosan Molecular Weight and Degree of Deacetylation

Chitosan molecular weight (MW) and DDA are key determinants of biological activity. High-MW chitosan typically exhibits enhanced film-forming ability and mechanical strength but may limit diffusion into microbial biofilms [16,17]. In contrast, low-MW chitosan and oligomers demonstrate improved solubility and permeability. The DDA governs the density of protonated amino groups; higher DDA generally enhances antimicrobial activity due to stronger electrostatic interactions with microbial membranes, though excessive charge density may increase cytotoxicity toward mammalian cells [19,20].

### 5.2. Curcumin Loading, Distribution, and Stability

Curcumin loading critically affects both antimicrobial performance and release kinetics. At low loading levels, insufficient concentrations may limit intracellular activity, whereas excessive loading can lead to aggregation and reduced bioavailability [11,12]. Uniform distribution within the chitosan matrix is essential for consistent activity. Encapsulation strategies, such as nanoparticle formation, can enhance curcumin stability and prevent premature degradation [49].

### 5.3. Crosslinking Density and Network Architecture

The degree of crosslinking influences mechanical properties, swelling behavior, and drug release kinetics. Increased crosslinking density enhances structural stability but may restrict the diffusion of curcumin [17]. Network architecture, whether hydrogels, films, or porous scaffolds, determines the accessibility of curcumin and material interactions with biological tissues. Highly porous structures facilitate fluid exchange and cellular infiltration, while dense matrices provide superior barrier properties.

### 5.4. Particle Size, Surface Charge, and Morphology

In nanoparticle systems, smaller particles exhibit enhanced penetration into biofilms, while larger particles may provide prolonged retention [49]. Surface charge, dictated by protonated amino groups, influences interaction with microbial membranes and cellular uptake. Positively charged particles exhibit stronger adhesion to bacterial surfaces, though excessive charge may increase non-specific cytotoxicity. Morphology also influences cellular interactions; for instance, nanofibrous structures can mimic extracellular matrix architecture to promote cell attachment.

### 5.5. Release Kinetics and Localization

Controlled release of curcumin is essential for maintaining therapeutic concentrations. Chitosan-based systems enable sustained release through diffusion-controlled and degradation-mediated mechanisms. These profiles can be tuned by adjusting crosslinking density and matrix composition [17]. The bioadhesive nature of chitosan ensures localized delivery, providing continuous exposure of the wound environment to therapeutic agents.

### 5.6. Balancing Antimicrobial Efficacy and Cytocompatibility

A critical challenge in the design of chitosan–curcumin systems is identifying an optimal balance between antimicrobial activity and biocompatibility. Parameters that enhance microbial inhibition—such as high charge density—may also increase host–cell toxicity. Systematic optimization is required to maximize microbial inhibition while preserving cell viability and promoting tissue regeneration.

### 5.7. Toward Rational Design of Multifunctional Systems

Establishing predictive relationships between material properties and biological outcomes is essential for the transition from empirical formulation to rational design. Key considerations include the selection of chitosan MW and DDA to balance penetration and activity, the optimization of curcumin loading for stability, and the engineering of network architecture to tailor release profiles (Table 1). Establishing clear structure–function relationships will be critical for advancing these systems into clinically viable therapies.

## 6. Clinical and Preclinical Evidence

### 6.1. Curcumin in Wound Healing

A growing body of clinical and experimental research supports curcumin as a biologically active agent capable of accelerating the healing of diabetic foot ulcers (DFUs) and chronic wounds. In a randomized, double-blind, placebo-controlled clinical trial, Mokhtari et al. demonstrated that oral curcumin supplementation significantly improved DFU wound closure while reducing systemic inflammation and oxidative stress, providing direct human evidence of therapeutic benefit [52]. Preclinical mechanistic studies further reinforce these findings: Cao et al. showed that curcumin promotes diabetic wound repair by suppressing microRNA (miR)-152-3p and activating the fibrillin-1(FBN1)/TGF-β pathway, thereby enhancing fibroblast activity, collagen deposition, and angiogenesis [53].

Curcumin-loaded nanocomposite hydrogel dressings markedly improve the healing of infected wounds through combined antimicrobial, anti-inflammatory, and pro-regenerative actions [54]. It exhibits broad wound-healing mechanisms—including the modulation of cytokines, macrophage activity, and extracellular matrix remodeling—across diverse chronic wound models [55]. These collective clinical and preclinical studies establish curcumin as a mechanistically robust and clinically promising therapeutic candidate for diabetic foot ulcers (DFU) management.

Gastric ulcers accompanied by acute or chronic bleeding pose a substantial risk of mortality, and achieving rapid hemostasis together with effective wound repair remains a major clinical challenge. To address this need, a thrombin-derived C-terminal peptide (TCP-25) was incorporated into two injectable, biocompatible carboxymethyl chitosan (CMC) hydrogels via a Schiff-base reaction. The resulting TCP-25–CMC hydrogels exhibit excellent adaptability to the harsh gastrointestinal environment, combining acid resistance with controlled degradation, sustained peptide release, preserved bioactivity, and strong bioadhesive properties. Both in vitro and in vivo studies demonstrate that the hydrogels achieve rapid hemostasis. In an ethanol-induced gastric ulcer model in rats, the formulation reduced gastric bleeding by 92% within 24 h—surpassing the efficacy of omeprazole, a standard clinical therapy. This enhanced performance arises from the synergistic actions of the CMC hydrogel matrix and the TCP-25 peptide, which together promote rapid hemostasis, inhibit bacterial growth, and accelerate gastric tissue repair in both acute and chronic bleeding ulcers. Collectively, these findings highlight a promising platform for clinical management of gastric ulcer bleeding and mucosal wound healing [56,57].

Curcumin is chemically unstable under physiological conditions and undergoes rapid hydrolysis, oxidation, and photodegradation. Within a wound, this instability is further exacerbated by reactive oxygen species, fluctuating pH, proteolytic enzymes, and abundant serum proteins, all of which accelerate its degradation or promote nonspecific binding to biological components. Consequently, free curcumin applied directly to a wound exhibits short local residence times, with tissue concentrations declining within hours. Although pharmacokinetic data specific to chronic wounds remain limited, studies in topical and burn-injury models consistently report rapid clearance and minimal systemic exposure. These limitations underscore the need for nanoformulations and polymeric carriers that can shield curcumin from degradation and prolong its local bioavailability. Curcumin-loaded nanoparticles, hydrogels, and film-forming systems have been shown to maintain detectable levels for 24–72 h while minimizing systemic absorption. Deng et al. [58] highlighted the substantial gains in stability and therapeutic performance achieved when curcumin is incorporated into engineered delivery systems. Complementing this, the classic kinetic study by Tønnesen and Karlsen [59] demonstrated that curcumin undergoes rapid breakdown in aqueous media under physiological and alkaline conditions, reinforcing the rationale for protective encapsulation strategies.

### 6.2. Chitosan in DFU Management

Chitosan has gained substantial attention as a multifunctional biomaterial for DFU management due to its intrinsic antimicrobial, hemostatic, and pro-regenerative properties. Its cationic amino groups interact electrostatically with negatively charged bacterial membranes, leading to membrane disruption and broad-spectrum antimicrobial activity, an essential feature in DFUs, where polymicrobial infection and biofilm formation impede healing [60].

At physiological pH, a fraction of chitosan’s amino groups remains protonated, giving the polymer a positive surface charge. This enhances its electrostatic attraction to negatively charged microbial membranes, promoting membrane destabilization, leakage of intracellular components, and impaired nutrient transport. The extent of protonation, together with molecular weight and degree of acetylation, strongly influences antimicrobial potency. These physicochemical parameters determine how effectively chitosan interacts with bacterial surfaces in the wound environment [61].

Beyond infection control, chitosan enhances early wound stabilization by promoting platelet adhesion and clot formation, while simultaneously stimulating fibroblast proliferation, keratinocyte migration, and extracellular matrix deposition [18].

Chitosan also exhibits immunomodulatory effects, reducing excessive inflammation and supporting the transition from the inflammatory to the proliferative phase of healing, a process often impaired in diabetic wounds [62]. Importantly, its versatility allows for fabrication into hydrogels, films, nanofibers, and nanocrystals, enabling controlled drug delivery and the synergistic incorporation of bioactive agents such as curcumin, growth factors, or silver nanoparticles [14,15]. Collectively, these attributes position chitosan as a cornerstone biomaterial capable of addressing the primary pathological barriers of DFUs: persistent infection, chronic inflammation, impaired angiogenesis, and delayed re-epithelialization.

### 6.3. Combined Curcumin–Chitosan Systems Versus Standalone Components

The therapeutic rationale for combining curcumin with chitosan in DFU management arises from the complementary and synergistic properties of both agents. Curcumin provides potent anti-inflammatory, antioxidant, and antimicrobial activities, but its clinical utility is limited by poor aqueous solubility, rapid degradation, and low bioavailability at the wound site. Chitosan, in contrast, offers intrinsic antimicrobial activity and wound-healing support through fibroblast stimulation, yet lacks the potent cytokine-modulating and ROS-scavenging effects required to counteract the chronic inflammatory environment of DFUs. When combined, chitosan acts as a stabilizing nanocarrier that enhances curcumin’s solubility, prolongs its local retention, and enables sustained release directly into the wound microenvironment.

This synergy is demonstrated in recent DFU-focused studies. Gómez-de la Cruz et al. [63] reviewed curcumin-loaded chitosan nanoparticles, highlighting their ability to overcome curcumin’s pharmacokinetic limitations while amplifying antimicrobial and anti-inflammatory effects, resulting in improved granulation tissue formation and accelerated DFU closure [63]. In a seminal preclinical study, Karri et al. incorporated curcumin-loaded chitosan nanoparticles into collagen–alginate scaffolds and observed significantly enhanced wound contraction, collagen deposition, and epithelial regeneration in diabetic models compared with curcumin or chitosan alone [64]. These findings underscore that the combination not only improves curcumin delivery but also leverages chitosan’s structural advantages to create a multifunctional dressing capable of addressing infection, inflammation, and impaired tissue regeneration simultaneously.

### 6.4. Curcumin/Chitosan with Antibiotics and Phages

Although curcumin and chitosan have each been explored in combination with antibiotics or bacteriophages, these strategies introduce mechanistic and translational challenges that limit their suitability for DFU management compared with curcumin–chitosan systems. Curcumin has shown the ability to potentiate antibiotic activity through membrane disruption, efflux pump inhibition, and the suppression of bacterial quorum sensing, with several studies demonstrating synergy against *S. aureus*, MRSA, and *A. baumannii* [9,29]. Curcumin can enhance the activity of selected antibiotics against clinically relevant multidrug-resistant bacteria, in many cases showing true synergism (e.g., with gentamicin and ciprofloxacin against MRSA) [65].

Chitosan has also been paired with antibiotics, where its cationic nature enhances membrane permeability and disrupts biofilms, improving antibiotic penetration [20,66]. However, this synergy is highly dependent on chitosan’s molecular weight, degree of deacetylation, and environmental pH values that vary widely and complicate clinical reproducibility. Furthermore, while chitosan and curcumin have been used to enhance phage therapy through layer-by-layer immobilization or encapsulation [67,68], chitosan’s strong cationic charge can reduce phage infectivity. Phage therapy itself faces regulatory, stability, and cold-chain constraints that limit its clinical deployment in DFU care [69]. In contrast, curcumin–chitosan systems avoid these limitations: the pairing is chemically compatible, inexpensive, and addresses the complexity of chronic wounds without the regulatory burdens associated with antibiotics or phages.

### 6.5. Curcumin and Chitosan Nanocomposites

Nanomaterial-based composites have been widely explored to overcome the poor solubility and instability of curcumin. Single-walled carbon nanotubes (SWCNTs) can load curcumin through hydrophobic and π–π interactions, improving stability; however, their biomedical use is constrained by metal contamination and intrinsic cytotoxicity [70]. Silica nanoparticles offer a safer, hydrophilic alternative where curcumin can be covalently linked to surface hydroxyl groups without altering its functional structure [71]. Poly(lactide-co-glycolide) (PLGA) nanoparticles provide controlled release and improved bioavailability, with a 50:50 lactide-glycolide ratio yielding up to nine-fold greater bioavailability than traditional formulations [72].

In parallel, chitosan-based nanoparticles (CS-NPs) represent an especially attractive platform due to their biocompatibility and intrinsic antimicrobial activity. CS-NPs enhance curcumin solubility, protect it from degradation, and enable sustained release while maintaining low cytotoxicity toward mammalian cells [73]. Chitosan can also form nanocomposites with metals (Ag, ZnO) or graphene derivatives to further improve antibacterial activity, though these hybrid systems require careful evaluation of dose-dependent cytotoxicity and long-term biocompatibility for medical applications [74,75].

### 6.6. Curcumin/Chitosan and Biosurfactants

Biosurfactants have recently emerged as promising adjuncts to curcumin- and chitosan-based systems due to their amphiphilic structure, intrinsic antimicrobial activity, and ability to enhance drug solubility. Microbial biosurfactants, including glycolipids and lipopeptides, exhibit potent antibacterial and antibiofilm properties, making them attractive candidates for chronic wound environments [15,16]. Surfactants can markedly improve curcumin solubility and dissolution by promoting amorphous dispersion formation and enhancing membrane permeability [76]. Surfactin, a cyclic lipopeptide, has been shown to form stable nanocapsules with curcumin, significantly increasing its bioavailability and cellular uptake [41].

Chitosan also interacts favorably with biosurfactants through hydrogen bonding and electrostatic interactions, enabling the formation of hybrid nanostructures with improved stability and antimicrobial performance [77]. These combinations have been applied in formulations where biosurfactant–chitosan systems demonstrated strong antimicrobial and antibiofilm activity with acceptable biocompatibility [78,79]. Collectively, the integration of biosurfactants with curcumin and chitosan offers a multifunctional platform that improves solubility and enhances antimicrobial potency in infected or biofilm-rich environments.

## 7. Chitosan Alone or in Composite Systems for Curcumin Delivery

Chitosan has been extensively investigated as a multifunctional carrier for curcumin delivery, owing to its favorable physicochemical and biological properties, including biodegradability, biocompatibility, and intrinsic antimicrobial activity. As emphasized in some comprehensive reviews [80,81,82], chitosan nanoparticles represent one of the most adaptable platforms for drug delivery, capable of improving the solubility, stability, and controlled release of hydrophobic compounds such as curcumin. The polycationic nature of chitosan facilitates strong electrostatic interactions with negatively charged biological membranes, thereby enhancing mucoadhesion and cellular uptake, as demonstrated in various delivery systems [83,84].

Curcumin, despite its broad-spectrum biological activities, suffers from poor aqueous solubility, rapid degradation, and limited bioavailability. Encapsulation within chitosan-based systems effectively addresses these limitations. For instance, chitosan nanoparticle-mediated delivery of curcumin significantly suppresses tumor growth [85], and improved in vivo performance using curcumin-loaded chitosan nanocapsules was demonstrated [86]. Similarly, enhanced bioavailability and tissue retention of curcumin following chitosan encapsulation underscores the clinical potential of such systems [87].

Beyond standalone chitosan carriers, numerous studies have explored composite formulations to further enhance delivery efficiency and functional performance. Chitosan–alginate systems [17,88] and chitosan–cyclodextrin complexes [89] enable improved encapsulation efficiency and pH-responsive release. Similarly, hybrid systems incorporating lecithin, liposomes, or polymeric matrices [90,91] enhance stability and bioavailability. Functionalization strategies, including quaternized chitosan [92] and amphiphilic grafting [93], further improve solubility and biological performance.

In addition, chitosan-based systems have been engineered in diverse formats, including nanoparticles, micelles, hydrogels, electrospun fibers, and composite scaffolds. For instance, chitosan/lignosulfonate micelles were prepared with enhanced antioxidant activity [94], whereas fabricated polycaprolactone–chitosan/curcumin fibers with controlled release and antimicrobial properties. More advanced co-encapsulation strategies [95] demonstrate the potential of chitosan systems to deliver multiple bioactives simultaneously, thereby enabling synergistic therapeutic effects.

To provide a structured overview of these diverse formulation strategies, Table 2 summarizes representative chitosan-based systems developed for curcumin delivery. The table highlights key aspects such as composition, delivery format, and functional outcomes, illustrating how different design approaches influence encapsulation efficiency, stability, and therapeutic performance. This comparative presentation underscores the versatility of chitosan platforms and facilitates the identification of structure–function relationships across various biomedical applications.

In summary, chitosan-based delivery systems provide a highly versatile and effective platform for overcoming the inherent limitations of curcumin, particularly its poor solubility and low bioavailability. Both standalone chitosan nanoparticles and composite formulations demonstrate significant improvements in stability, controlled release, and therapeutic efficacy across antimicrobial, anticancer, and wound-healing applications. Importantly, the integration of additional functional components enables the fine-tuning of physicochemical and biological properties, facilitating targeted and synergistic delivery. Future research should prioritize the standardization of chitosan characteristics and the optimization of formulation parameters to accelerate clinical translation.

## 8. Perspectives and Future Directions

Chitosan–curcumin systems have evolved from simple carrier–payload constructs into multifunctional bioactive platforms capable of addressing the intertwined challenges of infection, inflammation, and impaired tissue repair. Despite substantial progress, several critical knowledge gaps and translational barriers remain. Future research must prioritize the standardization of chitosan sources—including molecular weight, degree of deacetylation, and purity—as these parameters profoundly influence antimicrobial activity, cytocompatibility, and release kinetics. Likewise, curcumin stability and degradation pathways require deeper investigation under physiologically relevant conditions, particularly within chronic wound microenvironments characterized by fluctuating pH, proteases, and oxidative stress.

### 8.1. Antibiotic Synergy and Mechanistic Comparability

Chitosan has demonstrated synergy with multiple antibiotics through membrane disruption, charge-mediated interactions, and enhanced drug penetration [14,15]. Whether the curcumin–chitosan pair can achieve comparable or superior synergy remains an open question. Systematic studies should evaluate how curcumin’s intracellular targeting and redox modulation complement chitosan’s electrostatic and chelating mechanisms when co-formulated with antibiotics. Quantitative assays, such as checkerboard and time-kill analyses, will clarify additive, synergistic, or antagonistic effects across clinically relevant pathogens.

### 8.2. Antifungal and Polymicrobial Wound Applications

Beyond antibacterial activity, both curcumin and chitosan exhibit potent antifungal effects, particularly against *Candida albicans* and other opportunistic fungi [97,98,99]. Curcumin-loaded chitosan nanoparticles effectively disrupt mixed *C. albicans*–*S. aureus* biofilms, underscoring their potential for chronic wounds with polymicrobial infections [100]. Comparative studies with other nanocomposites and natural antimicrobials [101] could determine whether this dual-function system offers unique advantages in mixed bacterial–fungal environments. Understanding the balance between antifungal potency and cytocompatibility will be essential for safe clinical translation [102].

Chitosan exhibits direct antifungal activity and has been used clinically against *Candida* spp. It increases intracellular oxidative stress and permeabilizes fungal plasma membranes. Transcriptomic analyses in *Neurospora crassa* show that chitosan disrupts membrane homeostasis and oxidative metabolism, with specific membrane proteins and detoxification enzymes identified as key targets [103] (Lopez-Moya & Lopez-Llorca, 2016). Sensitivity varies among species; fungi with low polyunsaturated fatty acid content in their membranes, such as Pochonia chlamydosporia, show greater resistance.

Curcumin also displays antifungal activity by increasing membrane permeability, inhibiting hyphal formation, suppressing biofilm development, and interfering with efflux pump function, thereby enhancing susceptibility to antifungal drugs [104] (Martins et al., 2009). Given their complementary mechanisms, chitosan–curcumin systems are expected to provide broader antifungal coverage, including activity against fungal biofilms.

### 8.3. Formulation Complexity and Biocompatibility

Advanced delivery architectures—such as stimuli-responsive hydrogels, self-healing matrices, nanofiber scaffolds, and hierarchical multilayer films—offer opportunities for spatiotemporally controlled release and improved tissue integration. Integrating chitosan–curcumin systems with biosurfactants, metal nanoparticles, or crosslinking agents may further enhance antibiofilm and antifungal activity, but these hybrid systems demand rigorous evaluation of cytotoxicity, immunogenicity, and long-term biocompatibility. Future formulations should establish safe concentration thresholds and degradation profiles through comprehensive in vitro and in vivo testing [105,106,107,108], including the use of chitosan nanocrystals with antimicrobial activities [15].

### 8.4. Artificial Intelligence and Predictive Design

Artificial intelligence (AI) provides a transformative framework for optimizing chitosan–curcumin formulations. Machine-learning models can predict nanoparticle size, charge distribution, and release kinetics based on synthesis parameters, accelerating discovery and reducing experimental variability. AI-driven optimization has already improved chitosan nanoparticle biosynthesis and antibiofilm performance [109]. AI-guided nanoparticle delivery systems have enhanced curcumin’s targeted therapeutic potential in cancer models [110]. Therefore, integrating AI with high-throughput screening and molecular modeling could enable the rational design of composites with tailored antimicrobial, antifungal, and immunomodulatory profiles.

To achieve clinical adoption, future studies must address scalability, batch-to-batch reproducibility, and regulatory classification. Robust in vivo chronic wound models, including diabetic and ischemic wounds, are urgently needed to validate therapeutic efficacy. Ultimately, interdisciplinary collaboration among materials scientists, microbiologists, and clinicians will be crucial to transform these multifunctional systems into next-generation wound-healing biomaterials.

Three recent studies provide compelling evidence that combined chitosan–curcumin systems offer meaningful advantages for antimicrobial activity, biofilm disruption, and wound healing, particularly in infected or burn-injured skin. Although extensive literature exists on chitosan or curcumin individually, these works highlight the unique therapeutic synergy achieved when the two agents are co-formulated.

From an advanced technical viewpoint, microwave-modified chitosan–curcumin nanoparticles were specifically engineered for burn-wound repair, with microwave processing improving chitosan’s solubility and interaction with curcumin. This approach yielded nanoparticles with high encapsulation efficiency, enhanced stability, and sustained release profiles [111]. In vitro assays demonstrated strong antioxidant activity, broad antimicrobial effects, and accelerated fibroblast migration—key drivers of wound closure. In vivo burn-wound studies further showed markedly faster re-epithelialization, reduced inflammation, and improved collagen deposition compared with free curcumin or untreated controls. This work provides one of the clearest demonstrations that chitosan–curcumin nanoparticles can function as a regenerative platform for thermally damaged skin.

A second study prepared curcumin-loaded chitosan nanoparticles via ionic gelation and evaluated their wound-healing activity using physicochemical characterization, antimicrobial testing, and cell-based assays. The nanoparticles exhibited improved aqueous dispersibility, enhanced curcumin stability, and controlled release behavior. Antimicrobial testing confirmed activity against common wound pathogens, while scratch assays on L929 fibroblasts showed significantly accelerated wound closure relative to free curcumin [112]. These findings reinforce chitosan’s ability to enhance curcumin’s solubility and bioavailability while contributing to its own intrinsic antimicrobial and pro-healing properties, resulting in a synergistic therapeutic effect.

Equally important is the development of nano-curcumin-loaded chitosan/gum tragacanth nanofibers for infectious wound healing [113]. These hybrid nanofibers exhibited excellent mechanical strength, high porosity, and sustained curcumin release. Antibacterial testing demonstrated strong inhibition of *S. aureus* and *E. coli*, while in vitro wound-healing assays showed enhanced fibroblast proliferation and migration. In infected wound models, the nanofibers reduced inflammatory markers and supported tissue regeneration. This study highlights the versatility of chitosan–curcumin systems in solid-state wound dressings, extending their application beyond nanoparticles to functional biomaterial scaffolds.

Together, these three studies show that chitosan–curcumin combinations consistently outperform free curcumin in antimicrobial activity, antioxidant capacity, fibroblast migration, and wound closure.

The potential of chitosan–curcumin systems to eradicate mature biofilms, rather than merely inhibit early biofilm formation, also warrants discussion. Evidence indicates that these systems can reduce biomass and kill embedded cells within established biofilms. Curcumin-loaded chitosan nanoparticles significantly reduced the thickness of mono- and polymicrobial biofilms of *Candida albicans* and *Staphylococcus aureus* on silicone surfaces [100]. Curcumin–chitosan magnetic nanoparticles similarly demonstrated eradication activity against clinical MRSA isolates, with further enhancement when combined with oxacillin [114]. These findings suggest that chitosan–curcumin systems can penetrate and disrupt mature biofilms, although efficacy remains strongest during early biofilm development.

Combination strategies further improve outcomes as chitosan-functionalized metal nanoparticles generate ROS that enhance biofilm disruption [115]. Curcumin-mediated photodynamic inactivation is another effective modality, producing ROS that damage microbial cells and biofilm matrices [116,117]. These complementary mechanisms support the potential of chitosan–curcumin systems to target mature biofilms when integrated with ROS-generating or photodynamic approaches.

Across nanoparticle formulations, micellar systems, and electrospun dressings, the synergistic behavior of chitosan–curcumin platforms are consistently attributed to complementary mechanisms. Chitosan contributes mucoadhesion, intrinsic antimicrobial activity, and excellent biocompatibility, while curcumin provides anti-inflammatory, antioxidant, and anti-biofilm effects. Co-formulation also improves curcumin’s solubility, chemical stability, and release kinetics, resulting in enhanced biological performance compared with either agent alone.

Recent advances in nanocarrier engineering further support this synergy. Optimized nanocarrier architectures can overcome diffusion barriers and improve penetration into dense biological matrices, a principle directly relevant to chronic wound biofilms and exudate-rich environments [118]. In addition, precision nanoparticle design strategies, such as surface modification, size tuning, and controlled degradation, can enhance drug retention, cellular uptake, and therapeutic index [119]. These concepts align closely with the behavior of chitosan–curcumin systems, where chitosan’s cationic surface and curcumin’s hydrophobic core create a favorable platform for targeted delivery and sustained local activity.

Taken together, the studies cited in this section demonstrate that, although the literature spans diverse formulation types, the therapeutic potential of chitosan–curcumin systems are well supported by in vitro, in vivo, and mechanistic evidence [24,86,87,88,89,90,92,93,95,96,100,118,119].

## 9. Conclusions

Chitosan–curcumin systems represent a promising class of multifunctional biomaterials capable of addressing the complex pathophysiology of chronic and infected wounds. Their synergy arises from the integration of chitosan’s membrane-targeting, bio adhesive, and regenerative properties with curcumin’s intracellular antimicrobial, antioxidant, and immunomodulatory activities. This dual-function behavior enables simultaneous control of microbial viability, biofilm integrity, oxidative stress, and inflammatory signaling—features rarely achieved by conventional antimicrobials or passive delivery systems.

Despite these advantages, several challenges must be addressed to advance clinical translation. Curcumin’s poor solubility and instability necessitate optimized formulation strategies that balance enhanced bioavailability with cytocompatibility. Chitosan’s physicochemical variability, including molecular weight and degree of deacetylation, requires standardization to ensure reproducible biological performance. Hybrid systems incorporating biosurfactants, metal nanoparticles, or chemical modifiers offer enhanced antimicrobial activity but demand rigorous evaluation of long-term safety.

Future work should clarify the extent to which chitosan–curcumin systems can replicate or surpass the antibiotic synergy observed with other chitosan derivatives and assess their efficacy in polymicrobial and fungal wound models. Emerging AI tools provide new opportunities for predictive formulation design and the optimization of nanoparticle synthesis. Overall, chitosan–curcumin platforms hold significant potential as next-generation wound-healing materials, providing safe, scalable, and clinically effective solutions for managing complex wounds.

## Figures and Tables

**Table 1:** Representative antimicrobial activity of curcumin, chitosan, and chitosan–curcumin systems.

Material/System	Microorganism	MIC (µg/mL)	ZOI (mm)	Formulation	Key Observations	Ref.
Curcumin	*S. aureus*	125–250	10–14	Free compound	Moderate activity; limited by poor solubility	[9,29]
Curcumin	*E. coli*	200–400	8–12	Free compound	Lower activity against Gram-negative bacteria	[27]
Chitosan	*S. aureus*	500–2000	12–18	Solution (MW-dependent)	Strong membrane disruption	[20]
Chitosan	*C. albicans*	500–2500	12–20	Solution/film	Effective antifungal activity	[47]
Chitosan–Curcumin	*S. aureus*	25–100	18–25	Nanoparticles (~1:5–1:10 Cur:Chit)	Enhanced activity due to synergistic effects	[49]
Chitosan–Curcumin	*E. coli*	50–150	15–22	Nanoparticles/hydrogel (~1:5–1:10)	Improved penetration and delivery	[24]
Chitosan–Curcumin	*P. aeruginosa*	50–200	14–20	Composite systems	Significant biofilm disruption	[34]

**Table 2:** Representative chitosan-based delivery systems for curcumin: composition, formulation strategies, and functional outcomes.

System Type	Composition/Formulation	Delivery Format	Key Outcomes	Representative Studies
Chitosan nanoparticles (TPP crosslinked)	Chitosan + TPP + curcumin	Nanoparticles	Improved bioavailability, sustained release, anticancer activity	[85,95]
Chitosan nanocapsules	Chitosan + curcumin	Nanocapsules	Enhanced in vivo efficacy and stability	[86]
Chitosan–alginate composites	Chitosan + alginate + curcumin	Nanoparticles	pH-responsive delivery, improved bioavailability	[17,88]
Chitosan–cyclodextrin systems	Cyclodextrin–chitosan + curcumin	Nanoparticles	Controlled release, enhanced solubility	[89]
Chitosan–liposome hybrid	Liposomes coated with chitosan	Core–shell nanoparticles	Enhanced stability and release control	[90]
Electrospun fibers	PCL–chitosan + curcumin	Nanofibers	Controlled release, antimicrobial properties	[96]

## Data Availability

No new data were created or analyzed in this study.
